# Supplementary material for: Modelling the impact of effective private provider engagement on tuberculosis control in urban India
Source: Sci Rep. 2019 Mar 7;9:3810. doi: 10.1038/s41598-019-39799-7 (PMC6405912; doi:10.1038/s41598-019-39799-7)
Supplement: Supplementary file 1 — Supporting information [file 41598_2019_39799_MOESM1_ESM.docx]

**Modelling the impact of effective private provider engagement on tuberculosis control in urban India**

Supporting information

Nimalan Arinaminpathy^1^*, Sarang Deo^2^, Simrita Singh^2^, Sunil Khaparde^3^, Raghuram Rao^3^, Bhavin Vadera^3^, Niraj Kulshrestha^3^, Devesh Gupta^3^, Kiran Rade^4^, Sreenivas Achuthan Nair^5^, Puneet Dewan^6^

* Corresponding author: nim.pathy@imperial.ac.uk

*Author affiliations*

^1^ Department of Infectious Disease Epidemiology, Imperial College London, UK

^2^ Indian School of Business, Hyderabad, India

^3^ Central TB Division, Government of India, New Delhi, India

^4^ World Health Organization, India Country Office, New Delhi, India

^5^ Stop TB Partnership, Geneva, Switzerland

^6^ Bill and Melinda Gates Foundation, Seattle, USA

**1. Model specification**

The model is governed by the following equations (see table S1 for definitions of state variables, and table S2 for parameter definitions and sources). First, for the states prior to a TB patient’s first visit to a provider, we have:

$$\begin{matrix} \dot{U} & = & b-U\sum_{s} \lambda_{s}-\mu U \\ \dot{L_{s}} & = & \left( 1-f \right)\lambda_{s}\left[ U+\sum_{s} \left( L_{s}+R_{s}^{\left( hi \right)}+R_{s}^{\left( lo \right)} \right) \right]-\left( g+\mu\right)L_{s} \\ \dot{I}_{s} & = & f\lambda_{s}\left[ U+\sum_{s} \left( L_{s}+R_{s}^{\left( hi \right)}+R_{s}^{\left( lo \right)} \right) \right]+gL_{s}+\rho^{\left( hi \right)}R_{s}^{\left( hi \right)}+\rho^{\left( lo \right)}R_{s}^{\left( lo \right)}-\left( c+\sigma+\mu_{TB} \right)I_{s} \end{matrix}$$

where the subscript *s* denotes the infecting strain (with values 0,1 denoting drug-susceptible and drug resistant TB, respectively). The parameter *c* is the rate of careseeking, its inverse representing the average patient delay before first presentation for care.

Upon presenting for care, we assume that a proportion *p_r_* of patient visit a provider of type *r* (denoting the public sector; FQ providers; LTFQ providers; and chemists – see table S1). We have, for those awaiting diagnosis with provider type *r* and infected with strain s:

$$\begin{matrix} \dot{D}_{rs} & = & cp_{r}I_{s}+\gamma p_{r}\sum_{j} B_{js}-\left( d_{r}+h_{r}+\sigma+\mu_{TB} \right)D_{rs} \end{matrix}$$

As described in the main text, a patient-provider interaction may last days to weeks. This stage ends either when the provider finally offers a diagnosis (whether correctly for TB or otherwise), or when the patient leaves the provider, to seek care elsewhere. Here, we model these two endpoints through competing hazards of offering a diagnosis ($d_{r}$), versus the patient leaving the provider ($h_{r}$). As described below, these rates are estimated from the patient pathway surveys conducted in Mumbai and Patna ^1^.

We assume that a proportion $u_{r}$ of TB patients visiting provider type *r* successfully initiate TB treatment (the remainder constituting missed diagnosis as well as initial loss to followup, covered below). For those initiating first-line treatment, it is convenient to specify equations separately by drug-susceptible (*s* = 0) and drug-resistant (*s* = 1) status. Thus we have, for drug-susceptible TB:

$$\dot{F}_{r,0}=d_{r}u_{r}D_{r,0}-\left( \tau^{\left( FL \right)}+\delta_{r}+\alpha+\sigma+\mu\right)F_{r,0}$$

where $\delta_{r}$ is the per-capita rate of default from first-line treatment with provider type *r* and $\alpha$ represents the per-capita hazard of acquisition of multi-drug-resistance while on first-line TB treatment, only applicable to drug-sensitive TB. We assume that those defaulting from treatment are bacteriologically negative, but have an elevated risk of relapse, in comparison with those who have successfully completed treatment. The relevant compartments are discussed below.

For drug-resistant TB on first-line treatment, we have:

$$\dot{F}_{r,1}=d_{r}u_{r}{(1-v_{r})D}_{r,1}+\alpha F_{r,0}-\left( \tau^{\left( FL \right)}+\delta_{r}+{\sigma+\mu}_{TB} \right)F_{r,1}$$

where $v_{r}$ is the proportion of TB patients presenting to a provider of type *r* who undergo drug sensitivity testing at the point of TB diagnosis.

For second-line treatment (only for DR-TB), we have:

$$\dot{S}_{r,1}=du_{r}v_{r}D_{r,1}+\tau^{\left( FL \right)}w_{r}F_{r,1}-\left( \tau^{\left( SL \right)}+\mu\right)S_{r,1}$$

where $w_{r}$ represents the proportion of DR-TB patients with provider type *r* who are switched to second-line treatment after failing first-line treatment.

Next, the compartment *B* captures those patients who have dropped out of the care cascade and remain infectious, whether by failed diagnosis, loss to follow up, or failed treatment. We have, for *B*:

$$\dot{B}_{rs}=\left[ \left( 1-d_{r}u_{r} \right)D_{rs}+h_{r}D_{rs}+\left( 1-p_{q}^{\left( SL \right)} \right)\tau^{\left( SL \right)}S_{qrs} \right]-\left( \gamma+\sigma+\mu_{TB} \right)B_{rs}$$

Those who have recovered from disease include patients who have completed treatment; those who have defaulted from treatment; and those who have recovered spontaneously. We assume the latter two to have an elevated risk of relapse compared to the former, in the two years following recovery. Following this period, remaining recovered individuals stabilize in their relapse risk. Thus we have, for the ‘high’ and ‘low’ relapse risk compartments, respectively:

$\dot{R}_{0}^{\left( hi \right)}=\left[ \sum_{r} {\delta_{r}F}_{r,0}+\sigma(D_{r,0}+B_{r,0}) \right]+\sigma I_{0}-(\rho^{\left( hi \right)}+\mu+s)R_{0}^{\left( hi \right)}$

$\dot{R}_{0}^{\left( lo \right)}=\sum_{r} {\tau^{\left( FL \right)}F}_{r,0}-(\rho^{\left( lo \right)}+\mu+s)R_{0}^{\left( lo \right)}$

Finally, for the forces-of-infection $\lambda_{0},\lambda_{1}$ for DS- and DR-TB respectively, we have:

$\lambda_{0}=\beta\left[ \sum_{r} \left( I_{rs}+{\kappa B}_{rs} \right)+\kappa\sum_{r} D_{rs} \right]$,

and likewise for $\lambda_{1}$, but with $\beta_{MDR}$ in place of $\beta$.

**2. Patient pathways**

We adopted four different categories of provider: (i) those in the public sector (DOTS facilities); (ii) private chemists; (iii) private, ‘fully qualified’ (FQ) providers with qualifications in allopathic medicine; (iv) and private, ‘less-than-fully-qualified’ (LTFQ) providers with other medical qualifications, or none at all.

We used data from community-based patient pathway surveys, recently conducted in Mumbai (76 TB patients and 196 patient-provider interactions) and Patna (64 TB patients and 121 patient-provider interactions), and described in detail elsewhere ^1^. In brief, individuals in the community, who had been on TB treatment within the preceding 6 months, were administered an in-depth interview, to identify the sequence and types of providers that each patient visited before their TB diagnosis.

A patient’s contact with a given provider may last several days, sometimes weeks: this process ends either when the provider eventually makes a diagnosis, or when the patient drops out to visit an alternative provider. Here, we model this combination of behaviours using independent, competing exponential hazards with rates $r_{Diagnosis} and r_{Dropout}$,specific to the type of provider involved (public, FQ, LTFQ or chemist). Figure 1B shows the overall framework: for Mumbai and Patna separately. The rates are estimated from the data and their reciprocals give us the average time of diagnosis and the average time of dropout, respectively, for each type of provider. The probability of getting a diagnosis at a provider (whether a correct diagnosis or not) is equal to $\frac{r_{Diagnosis}}{r_{Diagnosis}+r_{Dropout}} ,$ and we estimate the accuracy of diagnosis of each type of provider from the data. We also model the role of different provider types in the careseeking pathway, in particular: the proportions of patients visiting each type of provider on the first careseeking attempt, and the corresponding proportions on subsequent visits, conditional on the type of provider last seen. 30 of the 196 patient-provider interactions in Mumbai, and 11 of the 121 patient-provider interactions in Patna, are such that the providers consulted are private, however, their qualifications, and hence their types (LTFQ/FQ), are missing. We let each missing provider type be represented by an unknown binary variable. Since the model parameters are specific to the provider type, the expression for the likelihood of the pathways data as a function of the model parameters also involves the missing binary variables. We use the iterative algorithm *Expectation Maximization* (EM) to obtain the maximum likelihood estimates of parameters. Each iteration involves two steps: *E- Step:* Finding the expectation of the log likelihood function, over the distribution of the missing binary variables conditioned on the observed data, under an initial estimate of the parameters, and *M- Step*: Maximizing the expectation of the log likelihood function to obtain a revised estimate of parameters. The revised estimate is then used as an initial estimate for the E-Step, and the process continues until the values of the maximum expectation of the log likelihood converge within a specified tolerance. The associated variance-covariance matrix of the estimates is approximated as the inverse of the observed Fisher Information Matrix, which is equal to the difference of the negative of the expectation of the Hessian matrix of the complete data log likelihood function, conditioned on the incomplete data and the expectation of the square of gradient of complete data log-likelihood function, conditioned on the incomplete data; evaluated at the final iteration of the EM algorithm.

For parameters related to the treatment cascade (the proportion of TB diagnoses initiating and completing treatment), we draw from a recent systematic review for the public sector ^2^. In the absence of systematic evidence for private providers, we incorporate plausible uncertainty distributions for these parameters (Table S2).

**3. Model calibration and propagating uncertainty**

We denote by $\theta$ the vector of input parameters, including $\beta,\beta_{MDR}, c,$and other model inputs subject to uncertainty. For a given country, and a given parameter set$\theta$, we initially simulated the model to equilibrium in the absence of the public sector (e.g. as in ref. ^3^) and MDR-TB, to capture the early history of the TB epidemic. Subsequently allowing population growth, we initiated the emergence and spread of DR-TB from 1980. We also captured the expansion of the public sector as a linear increase in $p_{0}$ during the years of RNTCP scale-up, i.e. from 1997 to 2006 ^4^. By combining these processes, we determined model projections for calibration targets (prevalence, ARTI and percent of incident TB cases being drug-resistant), assumed to apply in 2017.

To compare these model projections with data *D*, we defined the *posterior density* $\pi(\theta)$ as:

$\pi\left( \theta\right)\propto L\left( D | \theta\right).P(\theta)$,

where $L$ is the likelihood of the data *D* given $\theta$ and *P* is the joint prior distribution for $\theta.$ For *P*, we took independent uniform distributions over the ranges shown in table S2 (taking +/- 20% of the point values where no ranges are shown). The likelihood $L$ is constructed as follows.

We fitted a log-normal distribution to prevalence: in particular, we determined the mean and variance of this distribution in order for the 2.5^th^, 50^th^ and 97.5^th^ percentiles to match respectively the lower, mid and upper ranges of prevalence estimates. We write $F^{\left( Prev \right)}\left( \cdot\right)$ for the probability density thus obtained. Likewise, we write $F^{\left( ARTI \right)}\left( \cdot\right), F^{\left( pMDR \right)}\left( \cdot\right)$ for the inferred probability densities corresponding respectively to prevalence and the proportion of incident TB that is MDR in year *t*. Then we have, for the overall likelihood:

$$L\left( D|\theta\right)=F^{\left( Prev \right)}\left( Prev\left( \theta\right) \right)\cdot F^{\left( ARTI \right)}\left( ARTI\left( \theta\right) \right)\cdot F^{\left( pMDR \right)}(pMDR\left( \theta\right))$$

where, for example, $ARTI(\theta)$ is the simulated value of incidence in 2017 given parameters $\theta$, and likewise for the other functions of $\theta$ in the expression above. In practice we compute the logarithm of $\pi(\theta)$, thus taking the sum of the logarithms of each of the terms shown above.

With $\pi\left( \theta\right)$ thus defined, we sampled the posterior density using a Markov Chain Monte Carlo approach. In brief, this approach implements a random walk through the space of parameter values $\theta$to obtain an unbiased sample of the posterior density. We implemented the ‘adaptive’ MCMC algorithm first introduced by Haario et al ^5^, which incorporates a dynamic covariance matrix to adjust endogenously the scale of ‘jumps’ in proposals for each of the parameter values. For the set of parameter values thus obtained, we took every tenth element to reduce autocorrelation, thus yielding an ‘ensemble’ of parameters $\theta_{1},\theta_{2},\ldots$ This ensemble captures simultaneously the uncertainty in the parameter inputs, as well as in the calibration data. Then, to estimate uncertainty in given simulated outputs (e.g. in the reduction of incidence with a given coverage of intervention), we simulated this output $\Gamma_{i}$ for every $\theta_{i}$. We finally estimated uncertainty in $\Gamma_{i}$ by determining its 2.5^th^, 50^th^ and 97.5^th^ percentiles.


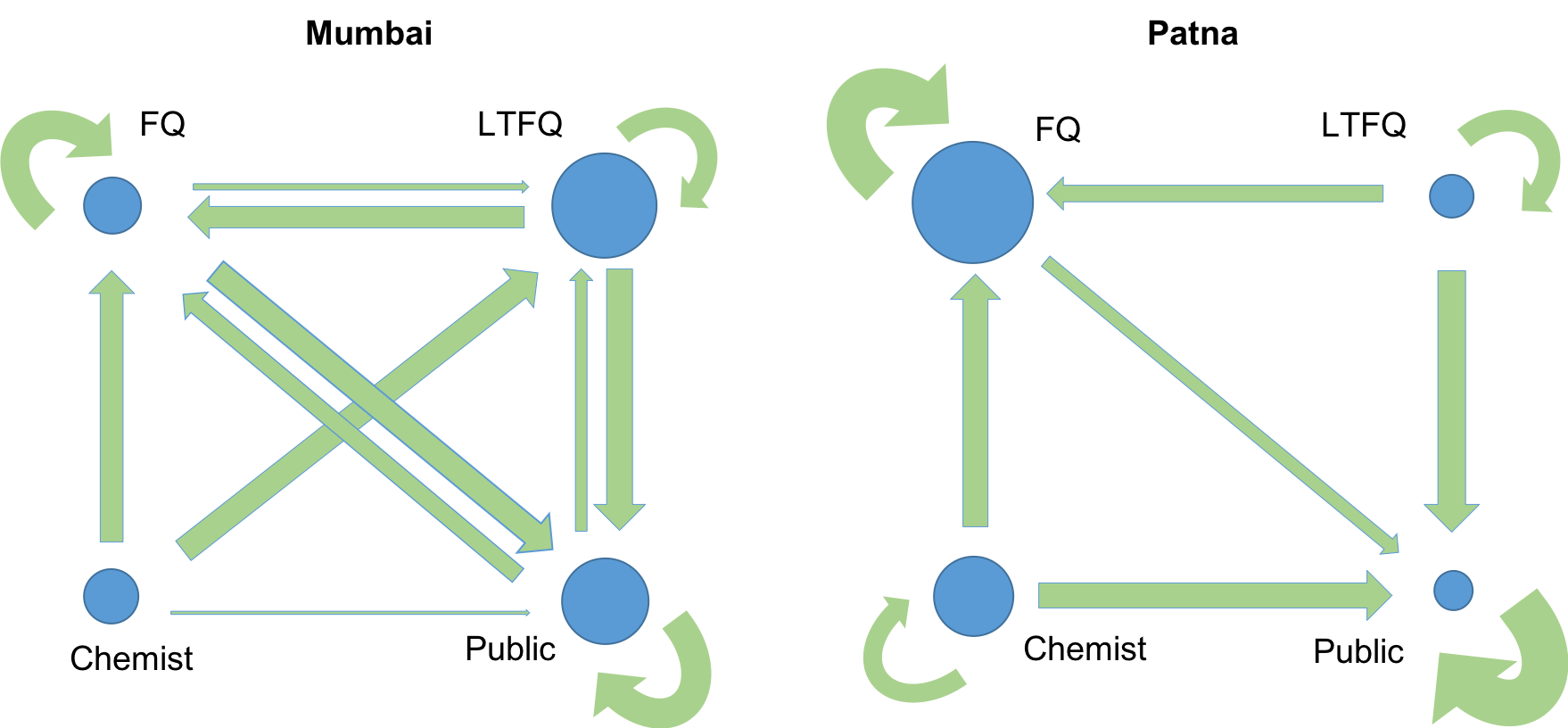


**Figure S1. Summary of the contrasting patient careseeking pathways in Mumbai and Patna.** Circle areas are proportional to the importance of providers as first point of patient contact (for example, patients in Patna tend to seek care first amongst fully qualified providers). Arrows denote how patients switch providers on subsequent visits, with arrow widths proportional to frequency.

**
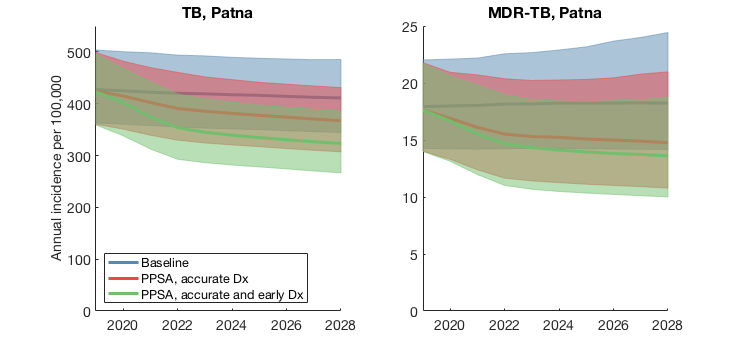
**

**Figure S2. Simulated impact of a PPSA in Patna**. As for Figure 3 in the main text, but for Patna. See Figure 3 caption for further details.


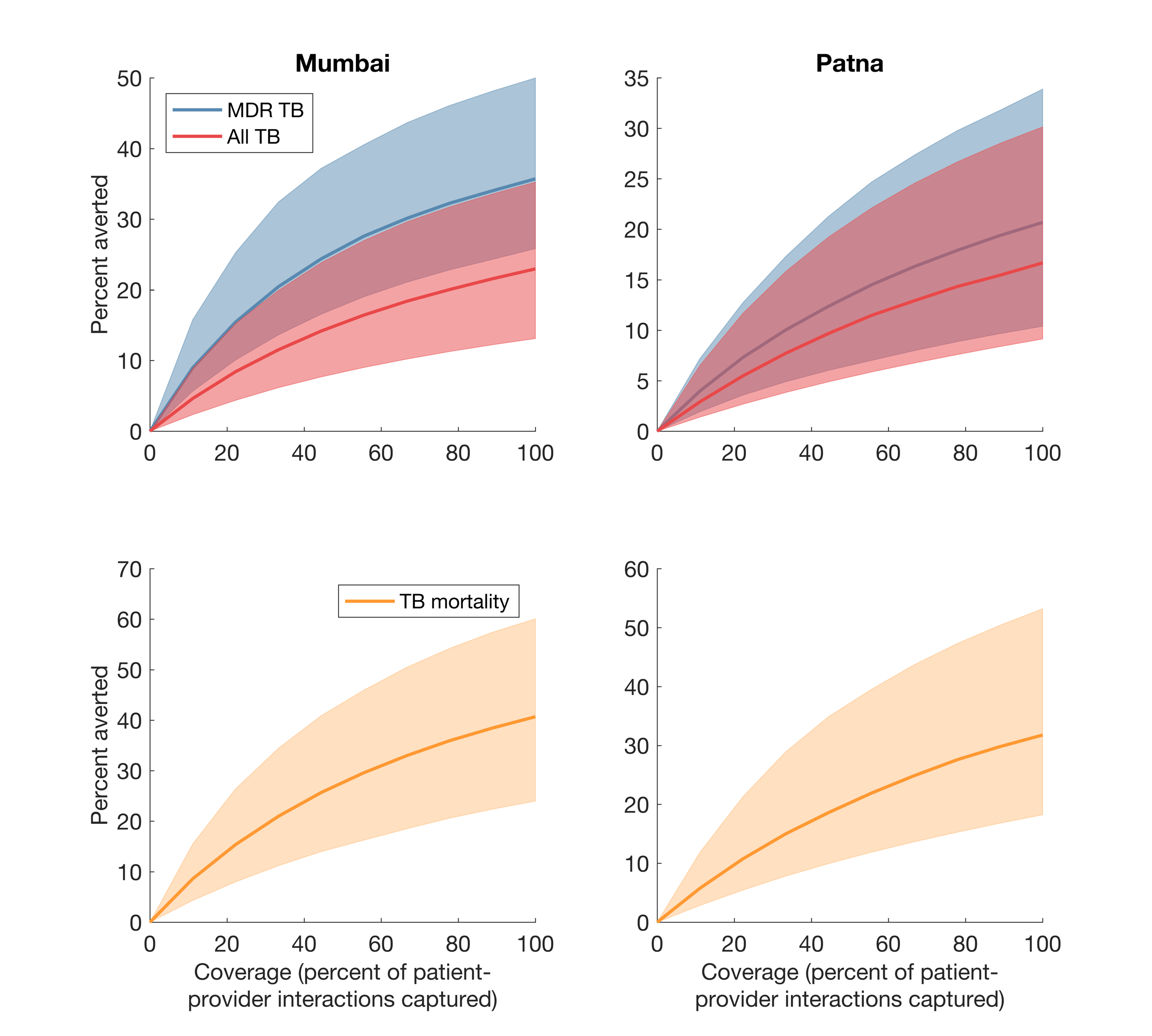


**Figure S3. Potential impact of a PPSA at different levels of coverage**, in Mumbai (left-hand column) and in Patna (right-hand column). Lines show central estimates, and shaded regions show 95% credible intervals.


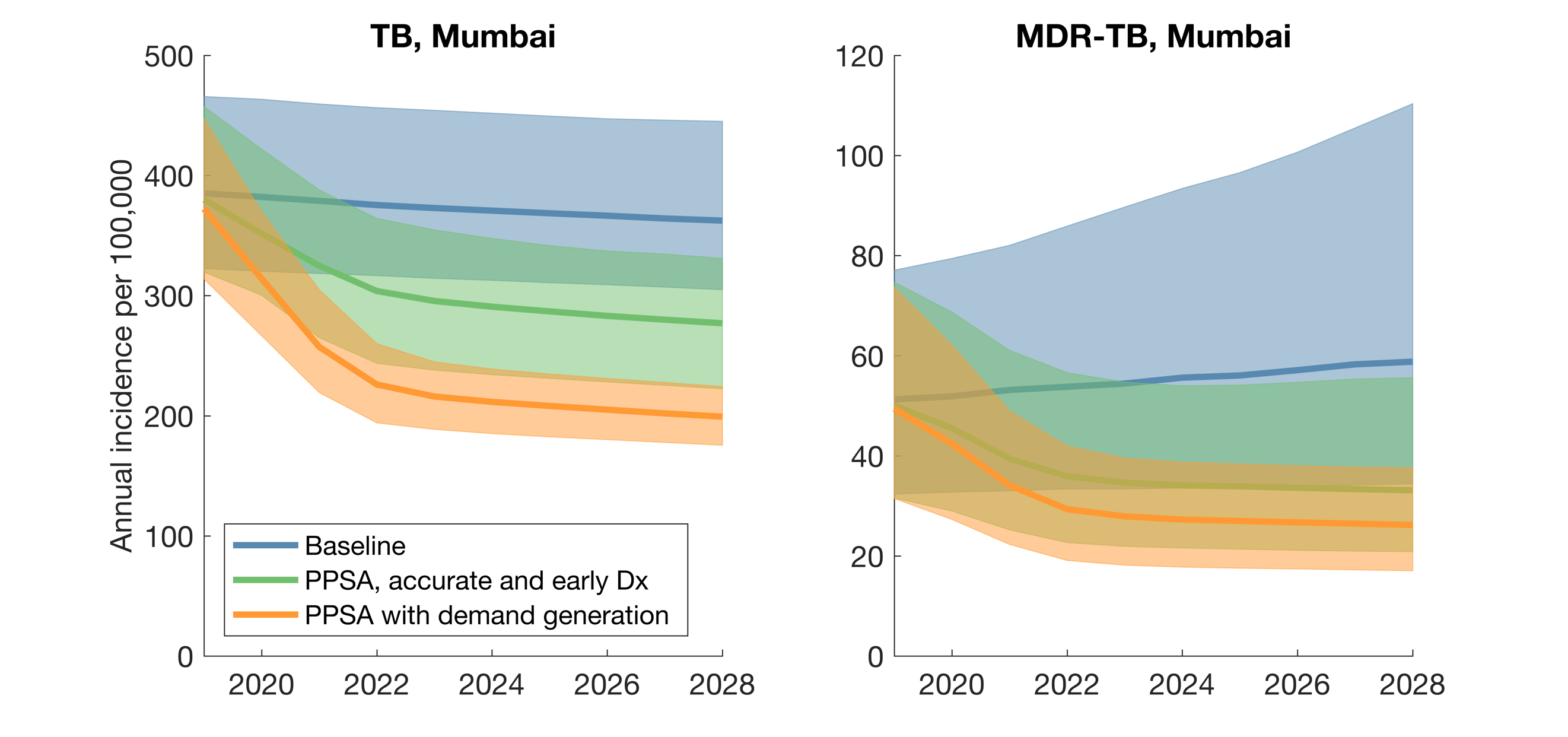


**Figure S4: Potential PPIA supplemented by demand generation** (yellow curve)**.** This provides the same scenarios shown in Fig.3 in the main text (a PPSA in Mumbai at 75% scale), but with ‘demand generation’ added for comparison. Uncertainty regions not shown, for clarity. Here, demand generation is assumed to bring about a 40% reduction in the patient delay. Such measures could involve lowering the barriers for access to care, or intensified case-finding. The impact shown here corresponds to a 37% (95% CrI 30.9 – 43.8%) reduction in cumulative incidence.

| Symbol | Meaning |
| --- | --- |
| *s* | Indicator variable for strain: *s* = 0, 1 respectively for DS- and DR-TB |
| $\boldsymbol{U}$ | Proportion uninfected |
| $\boldsymbol{L}_{\boldsymbol{s}}$ | Having *latent infection* with strain *s* |
| $\boldsymbol{I}_{\boldsymbol{s}}$ | Having *active disease* with strain *s,* but not yet presented for care |
| *r* | Indicator variable for provider type: *r* = 0 for the public sector; *r* = 1, 2, 3 respectively for FQ providers, LTFQ providers and chemists who are *not* engaged with the PPSA; and *r* = 4, 5, 6 for corresponding private providers who are engaged with the PPSA |
| $\boldsymbol{D}_{\boldsymbol{rs}}$ | Awaiting diagnosis with provider type *r* |
| $\boldsymbol{F}_{\boldsymbol{rs}}$ | Undergoing *first-line TB treatment* with provider type *r* |
| $\boldsymbol{S}_{\boldsymbol{rs}}$ | Undergoing *second-line TB treatment* with provider type *r* |
| $\boldsymbol{B}_{\boldsymbol{rs}}$ | Patients who have temporarily dropped out of care cascade, having visited provider type *r* |
| $\boldsymbol{R}_{\boldsymbol{s}}^{\left( \boldsymbol{hi} \right)}$ | Recovered with ‘high’ relapse risk (treatment defaulters and spontaneous recoveries) |
| $\boldsymbol{R}_{\boldsymbol{s}}^{\left( \boldsymbol{lo} \right)}$ | Recovered with ‘low’ relapse risk (following successful treatment) |

**Table S1. List of state variables used in the model.**

**Table S2. Input parameters and data used for the model.**

| Parameter | SYMBOL | | VALUE | Source/comments |
| --- | --- | --- | --- | --- |
| TB epidemiology |  |  |  |  |
| Annual risk of TB infection (ARTI) in urban slums as of 2015 |  | $\lambda$ | 2 – 3% | Gopi (2008)^6^ |
| TB prevalence in urban slums as of 2015 |  | *P* | 388 per 100,000 (233 – 543) | Baskaran (2015) ^7^ |
| Percent of incident TB cases that are MDR | Mumbai | *p*_MDR_ | 12% (8 – 16) | Assumption |
|  | Patna | *p*_MDR_ | 4% (3 – 5) | Assumption |
|  |  |  |  |  |
| TB natural history |  |  |  |  |
| Per-capita rate of reactivation of latent TB |  | *r* | 0.001 yr-1 | Horsburgh (2010) ^8^ |
| Proportion of infections undergoing rapid progression |  | $p_{fast}$ | 0.1 | Vynnycky (1997) ^9^ |
| Per-capita rate of relapse | Low relapse risk | $\rho_{lo}$ | 0.002 yr-1 (0.001 - 0.004) | Menzies (2009) ^10^ |
|  | High relapse risk | $\rho_{hi}$ | 0.02 yr-1 (0.01 - 0.04) |  |
| Per-capita mortality hazard | Non-TB | $\mu$ | 0.0152 yr-1 | World Bank estimates |
|  | TB cases | $\mu_{TB}$ | 0.089 yr-1 (0.33 - 1.21) | Tiemersma (2011) ^11^ (averaged across smear-positive and smear-negative TB), corresponding to 50% mortality in an average of 3 years |
| Per-capita rate of spontaneous cure |  | $\sigma$ | 0.089 yr-1 (0.33 - 1.21) |  |
| Anti-TB treatment (a, b) |  |  |  |  |
| Rate of completion of first-line TB treatment |  | $\tau_{1}$ | 2 yr-1 | Corresponds to a duration of 6 months |
| Rate of completion of second-line TB treatment |  | $\tau_{2}$ | 0.5 yr-1 | Corresponds to a duration of 2 years |
| Proportion of diagnosed TB cases initiating first-line TB treatment | Public | $p_{pu}^{FLinit}$ | 90% | RNTCP (2015) ^12^ |
|  | Private | $p_{pr}^{FLinit}$ | 0.6 (0.4 - 0.8) | Assumption |
| Proportion of diagnosed TB cases initiating second-line TB treatment | Public | $p_{pu}^{SLinit}$ | 90% | RNTCP (2015) ^12^ |
|  | Private | $p_{pr}^{SLinit}$ | -- |  |
| Proportion completing first-line TB treatment | Public | $p_{pu}^{FLcomp}$ | 85% | RNTCP (2015) ^12^ |
|  | Private | $p_{pr}^{FLcomp}$ | 0.6 (0.4 - 0.8)% | Uplekar (1998) ^13^ |
| Proportion completing second-line TB treatment | Public | $p_{pu}^{SLcomp}$ | 0.5 | RNTCP (2015) ^12^ |
|  | Private | $p_{pr}^{SLcomp}$ | -- |  |
| Per-capita rate of acquiring multi-drug resistance while on first-line treatment | Public | $m_{pu}$ | 0.01 yr-1 | Menzies (2009) ^10^ |
|  | Private | $m_{pr}$ | 0.05 yr-1 |  |
| Proportion of MDR-TB cases receiving drug susceptibility testing at point of TB diagnosis | Public sector | $dst_{pu}$ | 0.15 (0.05, 0.25) | Using data from national GeneXpert demonstration^14^ |
|  | Private (any type) | $dst_{pr}$ | 0 | Assumption (c) |

Notes:

1. As the pathway data does not have information about the quality of TB care, we have only partitioned these parameters by public vs private sector, assuming all 'private' parameters to apply to FQ, LTFQ providers alike.
2. See Table 2 for parameters relating to diagnosis, all inferred from the pathway surveys.
3. With the use of GeneXpert as a diagnostic tool, we assume this rises to 0.9 under a PPSA, with drug-resistant patients being referred to the public sector.

**Table S3. City-specific pathway parameters,** inferred from the pathway data

|  | |  | Public | FQ | LTFQ | Chemists |
| --- | --- | --- | --- | --- | --- | --- |
| Mumbai | | | | | | |
| *Proportion visited on initial consultation* | | | 0.30 (0.15, 0.47) | 0.13 (0.06, 0.22) | 0.45 (0.33, 0.57) | 0.12 (0.06, 0.19) |
| *Proportion visiting after leaving provider of type:* | Public | | 0.47 (0.24, 0.75) | 0.32 (0.14, 0.53) | 0.21 (0.06, 0.39) | 0 (0, 0) |
|  | FQ | | 0.46 (0.16, 0.81) | 0.43 (0.19, 0.72) | 0.11 (0, 0.3) | 0 (0, 0) |
|  | LTFQ | | 0.46 (0.23, 0.70) | 0.39 (0.20, 0.58) | 0.15 (0.03, 0.29) | 0 (0, 0) |
|  | Chemists | | 0.11 (0, 0.57) | 0.44 (0.17, 0.77) | 0.44 (0.17, 0.77) | 0 (0, 0) |
| *Probability of TB diagnosis per provider visit* | | | 0.94 (0.91, 0.98) | 0.93 (0.88, 0.98) | 0.75 (0.56, 0.94) | 0 (0, 0) |
| *Hazard Rate, provider offering diagnosis* | | | 0.087  (0.069, 0.106) | 0.074  (0.052, 0.096) | 0.035  (0.019, 0.051) | 0 (0, 0) |
| *Hazard rate, patient shopping* | | | 0.04 (0.03, 0.05) | 0.04 (0.03, 0.06) | 0.05 (0.03, 0.07) | 0.06 (0.05, 0.08) |
| Patna | | | | | | |
| *Proportion visited on initial consultation* | | | 0.063 (0, 0.29) | 0.61 (0.46, 0.77) | 0.08 (0, 0.20) | 0.25 (0.14, 0.36) |
| *Proportion visiting after leaving provider of type:* | Public | | 1 (1, 1) | 0 (0, 0) | 0 (0, 0) | 0 (0, 0) |
|  | FQ | | 0.24 (0.08, 0.42) | 0.76 (0.58, 0.95) | 0 (0, 0) | 0 (0, 0) |
|  | LTFQ | | 0.5 (0, 1) | 0.3 (0, 0.9) | 0.20 (0, 0.69) | 0 (0, 0) |
|  | Chemists | | 0.44 (0.22, 0.72) | 0.44 (0.22, 0.67) | 0 (0, 0) | 0.11 (0, 0.26) |
| *Probability of TB diagnosis per provider visit* | | | 1 (1, 1) | 0.95 (0.89, 1) | 0 (0, 0) | 0 (0, 0) |
| *Hazard Rate, provider offering diagnosis* | | | 0.18 (0.14, 0.23) | 0.10 (0.07, 0.14) | 0.0 (0, 0.17) | 0 (0, 0) |
| *Hazard rate, patient shopping* | | | 0.008 (0.006, 0.011) | 0.06 (0.04, 0.08) | 0.14 (0.04, 0.27) | 0.12 (0.09, 0.16) |

**Table S4. Model outputs for key transmission parameters**

|  | Infectivity, mean infections per year per case | | Mean duration, patient delay (months) |
| --- | --- | --- | --- |
|  | $\beta_{DS}$ | $\beta_{MDR}$ | *1/d* |
| Mumbai | 16.1 (9.7 – 28.8) | 12.8 (7.9 – 20.9) | 4.39 (1.64 – 9.57) |
| Patna | 15.1 (10.1 – 27.8) | 8.9 (5.3 – 17.5) | 5.24 (2.19 – 9.40) |

**References**

1 Mistry N, Rangan S, Dholakia Y, Lobo E, Shah S, Patil A. Durations and Delays in Care Seeking, Diagnosis and Treatment Initiation in Uncomplicated Pulmonary Tuberculosis Patients in Mumbai, India. *PLoS ONE* 2016; **11**: e0152287.

2 Subbaraman R, Nathavitharana RR, Satyanarayana S, *et al.* The Tuberculosis Cascade of Care in India’s Public Sector: A Systematic Review and Meta-analysis. *Plos Med* 2016; **13**: e1002149–38.

3 Mandal S, Chadha VK, Laxminarayan R, Arinaminpathy N. Counting the lives saved by DOTS in India: a model-based approach. *BMC Med* 2017; **15**: 47.

4 World Health Organization. A brief history of tuberculosis control in India. 2010.

5 Haario H, Saksman E, Tamminen J. An adaptive Metropolis algorithm. *Bernoulli* 2001; **7**: 223.

6 Gopi PG, Prasad VV, Vasantha M, *et al.* Annual risk of tuberculosis infection in Chennai city. *Indian J Tuberc* 2008; **55**: 157–61.

7 Dhanaraj B, Papanna MK, Adinarayanan S, *et al.* Prevalence and Risk Factors for Adult Pulmonary Tuberculosis in a Metropolitan City of South India. *PLoS ONE* 2015; **10**: e0124260–15.

8 Horsburgh CR, O'Donnell M, Chamblee S, *et al.* Revisiting rates of reactivation tuberculosis: a population-based approach. *Am J Respir Crit Care Med* 2010; **182**: 420–5.

9 Vynnycky E, Fine PE. The natural history of tuberculosis: the implications of age-dependent risks of disease and the role of reinfection. *Epidemiol Infect* 1997; **119**: 183–201.

10 Menzies D, Benedetti A, Paydar A, *et al.* Effect of duration and intermittency of rifampin on tuberculosis treatment outcomes: a systematic review and meta-analysis. *Plos Med* 2009; **6**: e1000146.

11 Tiemersma EW, van der Werf MJ, Borgdorff MW, Williams BG, Nagelkerke NJD. Natural History of Tuberculosis: Duration and Fatality of Untreated Pulmonary Tuberculosis in HIV Negative Patients: A Systematic Review. *PLoS ONE* 2011; **6**: e17601.

12 Revised National Tuberculosis Control Programme. TB India 2015 Annual Status Report. 2015; published online March 21. http://tbcindia.nic.in/showfile.php?lid=3166 (accessed Oct 5, 2016).

13 Uplekar M, Juvekar S, Morankar S, Rangan S, Nunn P. Tuberculosis patients and practitioners in private clinics in India. *Int J Tuberc Lung Dis* 1998; **2**: 324–9.

14 Sachdeva KS, Raizada N, Sreenivas A, *et al.* Use of Xpert MTB/RIF in Decentralized Public Health Settings and Its Effect on Pulmonary TB and DR-TB Case Finding in India. *PLoS ONE* 2015; **10**: e0126065.
